# Infection of Embryonic Callus with *Agrobacterium* Enables High-Speed Transformation of Maize

**DOI:** 10.3390/ijms20020279

**Published:** 2019-01-11

**Authors:** Dengxiang Du, Ruchang Jin, Jinjie Guo, Fangdong Zhang

**Affiliations:** National Key Laboratory of Crop Genetic Improvement and College of Plant Science and Technology, Huazhong Agricultural University, Wuhan 430070, China; ddx@mail.hzau.edu.cn (D.D.); jrc@webmail.hzau.edu.cn (R.J.); GJJ@webmail.hzau.edu.cn (J.G.)

**Keywords:** *Agrobacterium*, callus, maize, mixed enzyme solution, transformation

## Abstract

Several approaches have recently been adopted to improve *Agrobacterium*-mediated transformation of maize; however, about eight months of in vitro culture are still required to isolate transgenic plants. Furthermore, genetic transformation of maize depends on immature embryos, which greatly increases costs. Here, we report a method that ensures the competency of an embryogenic callus secondary culture under laboratory conditions for *Agrobacterium*-mediated transformation. Moreover, pretreatment of the cell wall with a mixed lytic enzyme solution prior to *Agrobacterium* infection, significantly improved transformation efficiency and stability. Average stable transformation efficiency was approximately 30.39%, with peaks of 94.46%. Expression and phenotypic analysis of the Rsc reporter gene were tested in the T_0_ generation of transgenic plants. Using this system, we successfully regenerated transgenic maize plantlets within three months of the emergence of the embryogenic callus. Additionally, we reduced somaclonal variation accompanying prolonged culture of maize cells in the dedifferentiated state, thus facilitating the molecular breeding of maize.

## 1. Introduction

Genetic transformation has been extensively used for the preparation of molecular research tools and improvements in breeding. Over the past three decades, the ability to accomplish heritable genetic transformation of plants has led to numerous crop applications that are essential for increasing global food supplies. Stable genetic transformation of major cereal crops, such as specific varieties of maize, rice, wheat, and barley, have changed from being solely laboratory vehicles for basic research to providing new varieties grown in large areas throughout the world [1,2,3,4,5,6,7,8,9,10,11]. Crucially, they can be used to alter the expression patterns of individual genes in a more precise and predictable manner than conventional breeding. The recent ability to perform genome editing in plants [12,13] has created an increased need for more efficient and genotype-independent plant transgenic biology.

Genetic transformation is considered one effective strategy to affect gene function in plants. In transgenic maize, it serves as an important tool for the development and study of germplasm, allowing for researchers to address fundamental biological questions. Transgenic maize plants were first obtained from protoplasts using an electroporation method [14]; then, different types of explants were used for transformation, such as protoplast [15,16,17], immature embryos [18,19,20,21,22,23], shoot apices [24,25,26,27], and immature embryo-derived calli [4,28,29]. Of the various modes of plant genetic transformation, including microprojectile bombardment, *Agrobacterium tumefaciens*-mediated transformation [21] is likely the most efficient and reliable. This preference is largely due to the advantages that the *Agrobacterium*-mediated T-DNA transfer process has over direct gene delivery systems, including a greater proportion of stable transgenic events than with the biolistic gun [18], the possibility of transferring larger DNA segments into recipient cells [30], and its high efficiency [22]. *Agrobacterium*-mediated transformation is a simple and cheap technology that inserts a lower copy number of transgenes, which helps to minimize gene silencing.

Maize transformation has been encumbered by several bottlenecks, including genotype and varietal dependence, explant sources, and specific cell culture dependence, as well as laborious, expensive, and time-consuming technologies. Recently, systems that are based on the embryogenic callus as the starting material for transformation have been developed. In some cereals, such as rice and maize, the embryogenic callus can be easily induced from embryos or the scutellum of immature embryos. This callus has also been transformed by biolistic and *Agrobacterium*-based techniques [3,31,32]. Although a few methods for the infection of maize embryonic callus with *Agrobacterium* have been developed, the transformation efficiency using immature embryo-derived maize varieties is still unsatisfactory, and only a limited number of maize cultivars have been genetically manipulated efficiently in this way. Thus, the method remains the limiting factor restricting successful transformation in maize [4]. *Agrobacterium*-based systems have been successfully used for the genetic transformation of maize, resulting in a greater degree of transgene stability than other methods [33,34]. The conversion rate is determined by the induction of the body cell embryo and preparation of competent cells. Optimization of experimental conditions is necessary to improve the number of competent cells and embryoid occurrence. A number of factors, such as bacterial strains, plasmids, tissue culture environment, media for explant culture, co-cultivation duration, use of acetosyringone, explant wounding, selective marker and vector, and competency of target plant tissues for infection [35,36,37,38,39], have been reported to affect the efficacy of *Agrobacterium*-mediated maize transformation. The progressive optimization of conditions for infection with *Agrobacterium* tumefaciens, such as the choice of vectors, the choice of strains, and tissue culture techniques, have made *Agrobacterium*-mediated transformation highly recommended for maize varieties with good tissue culture responses. However, when exposed to biotic and abiotic stress in adverse environments over long periods of time, plants gradually develop a series of complex defense reaction mechanisms, including intrinsic changes to the cell wall, a waxy layer that serves as a first line of defense against microorganisms, and a broad spectrum of resistance [40,41]. All of these changes affect the success of *Agrobacterium*-mediated transformation.

This report describes a new efficient system for the production of regenerable plants from a callus culture of maize immature embryos. Different treatments using standard binary vectors reveal that optimized culture conditions improved *Agrobacterium*-mediated stable transformation frequencies in maize cultivars in a genotype-independent manner.

## 2. Results

### 2.1. Agrobacterium Transformation Efficiency in Calli at Different Stages of Development

To determine the transformation level for different stages of maize immature embryos and calli that are produced from such embryos, we first evaluated instantaneous conversion efficiency and conversion rate using different receptor materials and a green fluorescence reporter. Results showed no significant difference in instantaneous conversion efficiency of the HiII callus at different induction stages (Table 1). The average instantaneous conversion efficiency with immature embryos was 5.3% for three times experiment. The average instantaneous conversion efficiency was 4.7% using type I callus as the receptor and the conversion rates were 7%, 3.9%, and 3.2%, respectively. The average instantaneous conversion efficiency with dense type II callus was 5.9%, the transformed callus were obtained at 5.94 g, 5.67 g, and 4.32 g from 90 g receptor material, respectively. Obtained 4.23 g (4.7%), 5.22 g (5.8%), 5.49 g (6.1%), and average 5.5% instantaneous conversion efficiency for loose type II callus.

Instantaneous into the receptor, choose different processing materials for successive transfer culture further, after three months of training to use fluorescent phenotype on DR-46B Dark Reader transilluminator on the screening of 4.72% in the final conversion structure loose type II callus and 0.69% of the conversion efficiency in the structure of type II callus. After three months of succession, the conversion rates of 0.05% and 0.08% in immature embryos and type I callus were obtained, respectively (Table 2).

**Figure 1:**
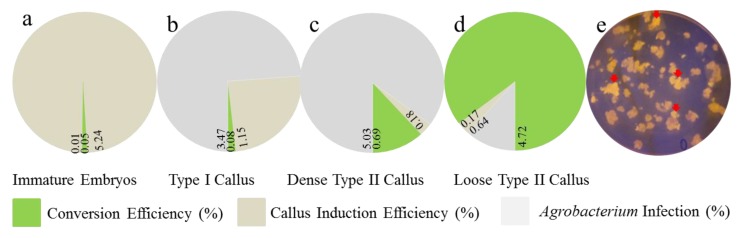
*Agrobacterium* transformation efficiency based on different states of the callus. (**a**) Conversion efficiency and loss rate for callus induction and *Agrobacterium* on immature embryos. Columnar volume represents material that is transiently transformed; The green plate represents conversion efficiency rate; Brown plate represents the callus loss caused by callus induction. The grey plate representsrepresents the callus loss caused by *Agrobacterium* infection; (**b**) Conversion efficiency and loss rate for callus induction and *Agrobacterium* on Type I callus. The green plate represents conversion efficiency rate; Brown plate represents the callus loss caused by callus induction. The grey plate representsrepresents the callus loss caused by *Agrobacterium* infection; (**c**) Conversion efficiency and loss rate for callus induction and *Agrobacterium* on Dense Type II callus. The green plate represents conversion efficiency rate; Brown plate represents the callus loss caused by callus induction. The grey plate representsrepresents the callus loss caused by *Agrobacterium* infection; (**d**) Conversion efficiency and loss rate for callus induction and *Agrobacterium* on Loose Type II callus. The green plate represents conversion efficiency rate; Brown plate represents the callus loss caused by callus induction. The grey plate representsrepresents the callus loss caused by *Agrobacterium* infection; and, (**e**) Callus phenotype on DR-46B Dark Reader transilluminator in darkness. Callus with green fluorescence are indicated by the red arrow.

Even though not all transformed cells were guaranteed to produce transformed calli, 3–4 cells appeared sufficient. Accordingly, numerous embryogenic transformed calli could potentially be obtained in six months. Conversion efficiency after six months of selection was affected by callus induction efficiency, *Agrobacterium* infection, and the transfer of immature embryos or calli to recovery medium for subculture after transformation. Conversion efficiency and loss of different receptor materials were statistically analyzed by green fluorescence (Figure 1e). As shown in Figure 1a, only 0.05% of transformed immature embryos obtained callus allowed for differentiation and regeneration of plants, for that 99.06% immature embryos infected by *Agrobacterium* cannot induce the callus. At the same time, in The use of type I calli as receptors for transformation not only failed to induce type II calli, but also reduced the rate of successfully transformed calli to 2.44% of resistant specimens with a 73.83% *Agrobacterium* infection rate (Figure 1b). A similar transformation rate was obtained for more closely structured type II and type I calli. When considering the infection rate of *Agrobacterium* and an induction rate of the embryonic callus of 73.83%, the final conversion rate was 0.69% (Figure 1c). In particular, when loose type II calli were used as receptors for *Agrobacterium*-mediated infection and transformation, the loose structure facilitated dispersion, and an infection rate of only 11% yielded a transformation rate of 4.72% and a final conversion rate >80% (Figure 1d). Overall, the latter required no more than 3–4 months of succession screening.

### 2.2. Optimization of the Agrobacterium Transgenic System on Calli

**Figure 2:**
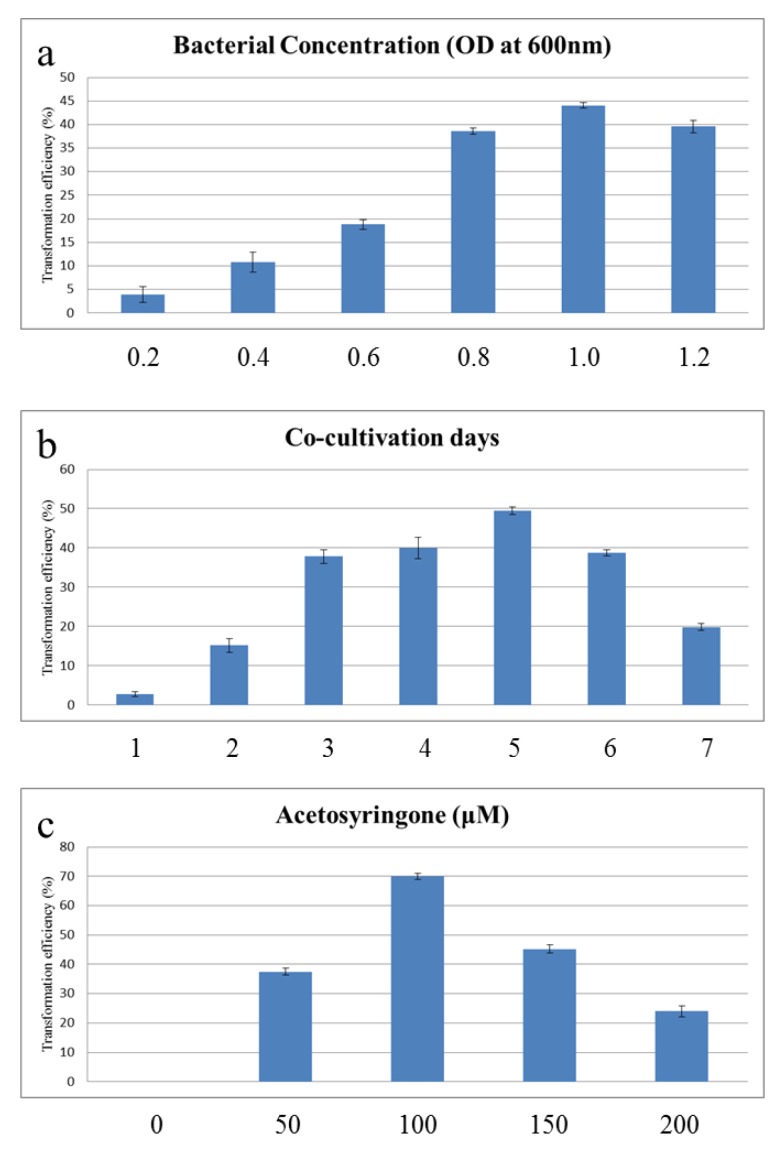
Effect of different parameters of the *Agrobacterium* transgenic system on transformation of maize callus. (**a**) Effect of bacterial culture density; (**b**) Effect of co-cultivation period; and, (**c**) Effect of acetosyringone concentration.

Here, we looked at the effect of bacterial density on transformation efficiency of the maize callus. The frequency of the reporter’s transient fluorescence expression in the six different bacterial cultures varied as a measure of optical density at 600 nm (OD_600_). Based on measurements of OD_600_ at 0.2, 0.4, 0.6, 0.8, 1.0, and 1.2, maximum transformation efficiency (44.06%) was observed at OD_600_ 1.0 (Figure 2a). Days of co-culture were tested as another parameter affecting transformation efficiency. No transformation was observed on day 1 of co-cultivation. Maximum transformation efficiency (49.44%) was observed after five days of co-cultivation (Figure 2b). To evaluate the effect of acetosyringone on final transformation efficiency, different concentrations of acetosyringone (0, 50, 100, 150, and 200 μM) were used at the time of co-culture. Transformation efficiency augmented with increasing concentrations of acetosyringone, reaching a maximum efficiency (69.98%) at 100 μM acetosyringone (Figure 2c).

### 2.3. Effect of Pretreatment with a Mixed Lytic Enzyme Solution

We tested how different pretreatment conditions influenced the frequency of transient expression and stable *Agrobacterium* transformation in maize; the results are shown in Table 3 and Table 4. Two treatment methods, including different concentrations of a mixed enzyme solution and different processing times, as well as two types of processing conditions were examined by detecting fluorescence expression of the reporter gene.

The explants were pretreated with a mixed enzyme solution at concentrations of 0.000, 0.010, 0.020, 0.030, 0.040, and 0.050 g/mL to investigate the effect of enzyme concentration on callus rate and stable transformation frequencies. The callus rate decreased from 73.19% to 18.47% when the enzyme concentration increased from 0.000 g/mL to 0.050 g/mL (Table 3). Maximum stable transformation frequency, as determined by fluorescence expression (37.48%), was obtained at 0.030 g/mL, but decreased thereafter. Different processing times (0, 3, 6, 9, 12, and 15 min) were tested to optimize the pretreatment period for different concentrations of enzyme solution. The explants were selected for three weeks. The callus rate declined sharply after prolonged pretreatment (Table 3). Callus conversion peaked at 38.33% after 9 min, but decreased thereafter. At 9 min, the permeability of the maize callus’ surface was highest and the number of transformed cells increased significantly. Overall, taking into account both, embryonic calli yield and stable conversion efficiency, the optimum processing conditions were achieved with an incubation of 9 min at an enzyme concentration of 0.030 g/mL. These conditions effectively improved *Agrobacterium*-mediated transformation efficiency.

A combined assessment of the effect of pretreatment time and enzyme concentration revealed a similar trend as in the two separate sets of experiments. Conversion efficiency was greatest after a first increase in either parameter, but it declined when either time or concentration were further increased (Table 4). The processing time could be extended to compensate for a low concentration, and high lytic enzyme levels required less time to achieve the goal. Again, the highest frequency of fluorescence expression was achieved with a 9-min treatment at a concentration of 0.030 g/mL. High concentrations of enzyme solution significantly reduced the proportion of stably transformed calli, which was accompanied by a decline in the cell death ratio. A similar trend was observed with single concentrations at different time points, whereby an increase in enzyme concentration, caused a decline in the transformed callus rate. The lowest rate of stably transformed calli was obtained after 15 min of treatment. Thus, lytic enzyme pretreatment conferred Hi-II maize calli with higher *Agrobacterium*-mediated transformation efficiency.

### 2.4. Transformation and Regeneration of Putative Transformed Plants

**Figure 3:**
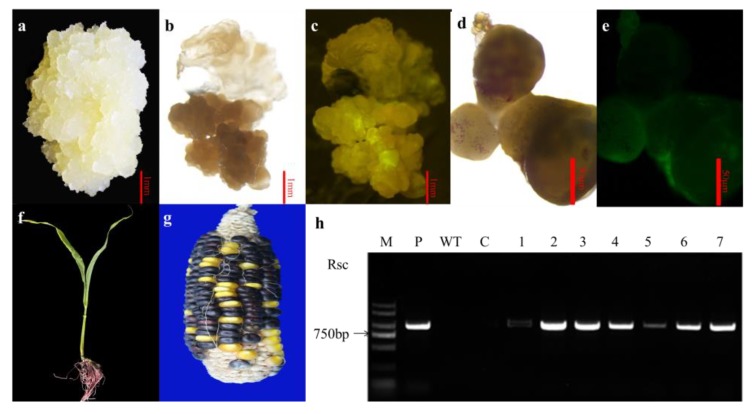
Regeneration of transgenic maize lines from immature embryogenic calli, and molecular and phenotypic identification of transformants. (**a**) Callus derived from an immature embryo and used as transformation receptor; (**b**) Fluorescence-positive calli grown on selection medium under the white light; (**c**) Fluorescence-positive calli phenotype was detected on the DR-46B Dark Reader transilluminator; (**d**) Multiple green spots were visible on the embryogenic calli in regeneration medium; (**e**) Fluorescence phenotype was detected with a fluorescent protein macro detector set; (**f**) Regeneration of putative transgenic plants; (**g**) Rsc-positive seeds of the regenerated plant; (**h**) PCR analysis showing positive transplants. M, BM2000 DNA marker; P, Plasmid DNA; WT: Wild type control; C: Transgenic negative control; lanes 1–7, different plants from different transformants.

In our final experiment, calli were obtained following a second round of selection (Figure 3a), with new callus growth appearing after approximately 40 days. After three rounds of selection, actively growing calli in selection media were positive for green fluorescence (Figure 3b,c). Following selection based on green fluorescence, the calli were transferred to the regeneration medium. Multiple green spots were visible on the embryogenic calli after 10 days in regeneration medium. The green sectors further differentiated into shoot meristems. Multiple shoots that were obtained following incubation in regeneration medium did not elongate in the same medium (Figure 3d,e). After transfer to rooting medium, the multiple shoots exhibited rapid shoot elongation, and rooting was observed (Figure 3f). Plants flowered normally and produced viable seeds. The seeds of the regenerated plants were *Rsc*-positive and did not show any detectable abnormalities in morphology or growth characteristics (Figure 3g). PCR analysis of genomic DNA revealed the presence of transgenes in the putative transformants of T_0_ maize plants. Expected *Rsc* fragments of 914 bp were detected in the T_0_ plants but not in non-transformed plants (Figure 3h).

## 3. Discussion

The development of transgenic technology provides a new way for crop genetic and breeding work. Through transgenic technology, reproductive isolation can be bypassed to the greatest extent, which provides the possibility of free exchange of high-quality genetic resources between different species and it also brings the possibility of breakthrough breeding work [42]. Maize transgenic manipulation is a complex process that involves the preparation of transformed receptor cells (transformed receptors), exogenous gene introduction (transformation methods), screening and identification of transformed cells (selection and application of screening markers), regeneration of transformed cells, and a series of functional expression verification processes [43].

High transformation frequency of maize mediated by *Agrobacterium* has been reported as the method of choice for the delivery of exogenous genes into the maize genome. Transgenic maize operations mediated by *Agrobacterium* is limited by the type of the recipient material limited, immature embryos were thought to be almost the only receptor material for *Agrobacterium*-mediated maize transgenes, although their applications have been limited [44,45]. Specifically, transformation efficiency using immature embryo-derived maize calli remains unsatisfactory. The identification of receptor material is an important content of maize transgenic research and application.

The embryonic callus has been used for genetic transformation in maize, but particularly as a receptor for gun-mediated transformation [4,46,47]. Embryonic callus, as a transgenic receptor material, should have the following advantages: it has a high ability of receiving exogenous DNA and genetic stability, and exogenous genes can be expressed and inherited in plant genomes through stable integration through transformation. As a receptor material, embryonic callus is sensitive to the screening agent and it can effectively distinguish transformed cells from non-transformed cells in the medium containing the screening agent. In addition, embryonic callus has a more stable source when compared with young embryos, which can be obtained stably through tissue culture work in the laboratory and can be continuously supplied for transgenic operation and application, thus successfully avoiding seasonal restrictions of immature embryo materials, greatly reducing the difficulty and cost of maize transgenic operation. In this study, immature embryos, type I callus, dense type II callus, and loose type II callus were selected as receptor materials to conduct *Agrobacterium*-mediated transgenic operations, and the instantaneous conversion efficiency and the yield of positive callus under the same transformation conditions were calculated, respectively. The experimental results show that no significant instantaneous efficiency difference between different stages callus, the conversion efficiency were 0.99% (immature embryo), 1.72% (type I callus), 0.69% (compact type II callus), and 4.72% (loose type II callus), separately. The difference between the conversion efficiency and the instantaneous conversion efficiency is mainly caused by the callus induction ability and the resistance of *Agrobacterium* infection. For immature embryos, the loss was mainly due to the fact that a large number of transformed cells could not induce embryonic callus, and the loss rate was as high as 98.87%. Type I callus loss was 24.47% and 73.83%, respectively, after transformation, resulting from un-inducible in good condition and residual *Agrobacterium* infection in the interstitial space. In loose type II callus with a conversion rate of 85.82%, dense type II callus lost 85.25% of transformed cells due to residual *Agrobacterium* infection between the interstitial spaces. In addition, the application of embryonic callus as the receptor material in this study can significantly simplify the transformation process, as compared with immature embryos. Maize transgenic operations mediated by *Agrobacterium* can be completed within three months, and it has the advantage of stable and extensive source of embryonic callus, similar results were reported previously regarding monocot transformation [48].

As several factors can affect *Agrobacterium*-mediated transformation efficiency [19,20,21,49,50,51], transformation frequency remains low relative to standard binary vector systems, even with optimized medium components; further improvements in transformation efficiency and transformation conditions are necessary. To establish an efficient transformation protocol, modulation in the concentration of acetosyringone, co-culture period, and in bacterial growth needs to be optimized. The addition of acetosyringone in the co-cultivation media, which acts as an inducing agent for T-DNA transfer into the host cell, significantly increases the efficiency of rice transformation. In our experiment, the transformation efficiency was enhanced with increasing concentrations of acetosyringone, with the maximum transformation efficiency (69.98 %) at 100 μM of acetosyringone. Our results are in agreement with Du et al., who reported a final concentration of 100 μM for the enhanced transformation efficiency in maize [52]. Therefore, the addition of acetosyringone appears to be essential for improving transformation frequency, although its exact concentration in the co-culture medium may vary between different plants [53]. Different days of co-culture period and bacterial cultures of ODs were also used as a parameter to investigate the transformation efficiency. In the present study, we observed that the use of a lower concentration (OD1.0) of *Agrobacterium* culture suspended and five days of co-culture on media are optimum for transformation. It may possibly be because of the reduced damage to explants during *Agrobacterium* infection, which results in less phenolic production and better recovery of callus during the selection. Similar to our study, the influence of the concentrations of acetosyringone, co-culture period and cell density on the efficiency of T-DNA transfer has been studied in the transformation of many plant species [54,55,56].

Tissue culture of receptor cells and reproduction of transformed cells are key links for maize transgenic operation, and the receptor is considered to be the most important basic condition for plant transgenic [47]. Unlike bacterial factors, the role and identity of host factors involved in the transformation process have remained obscure for nearly a century; only recently, have we begun to understand how *Agrobacterium* hijacks host cellular processes during transformation [57,58,59,60]. Various stress reactions are elicited by pretreatment with lytic enzymes, which cause damage to the cell wall of maize cells. These stressful conditions create competent cells, and allow *Agrobacterium tumefaciens* to recognize and attach to the surface of maize cells through loose cell walls. If more bacteria attach to the cytomembrane of the explant, their chances of entering the cells increase. In addition, because the cells are wounded, the trauma makes the conditions more suitable for transformation. Based on these results, the pretreatment of the callus surface with an enzyme solution before infection improved the transformation frequency because maize calli were more competent for *Agrobacterium*-mediated transformation. The percentage of embryonic calli could be gradually reduced from 73.19% to 18.47% and decreased with increasing concentrations of enzyme or by prolonging the pretreatment, whereby the callus became similar to a wet sponge, i.e., transparent and more loose. Overall, while taking into account both, embryonic calli yield and stable conversion efficiency, the optimum processing conditions were achieved with an incubation of 9 min at an enzyme concentration of 0.030 g/mL. These conditions effectively improved *Agrobacterium*-mediated transformation efficiency.

To produce transgenic events, a short pretreatment was the most stable method for improving genetic transformation efficiency [61,62]. This finding suggests that a moderate concentration of enzyme solution can stimulate cell division, promote callus growth, and improve *Agrobacterium* infectivity and transformation efficiency. However, excessively high enzyme levels can seriously damage callus tissue, thus resulting in a higher conversion rate. In addition, longer pretreatment times significantly reduced the proportion of embryonic connective tissue, analogously to pretreatment with high levels of enzyme. This finding might indicate that a longer lytic activity resulted in more competent cells with seriously damaged cell walls.

## 4. Materials and Methods

### 4.1. Plant Material

**Figure 4:**
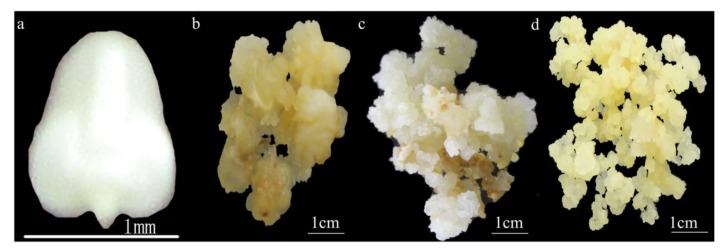
Immature embryos of maize Hi-II hybrid maize (A188*B73 origin) and different states of callus induced from the immature embryos in Induction process. (**a**) Immature embryos were isolated at 1.5–2.5 mm in length; (**b**) Type I callus with clumps of nonpolar cells with a smooth surface and a hard shell, was generated at the edge of the immature shield after 20–40 days on induction medium; (**c**) Type II callus by bright color, large granulation and hardness surface was generated after 60–90 days on induction medium; and, (**d**) Type II callus characterized by bright color, loose structure, granular shape, dry surface, and rapid growth was generated after 60–90 days on induction medium.

F_2_ immature embryos of maize Hi-II hybrid maize (A188*B73 origin) were used as the initial material and were cultivated on an experimental farm at Huazhong Agricultural University in Wuhan, China. Following self-pollination at approximately 10–14 days, the ears were harvested when the immature embryos were 1.5–2.5 mm in length (Figure 4a). To sterilize their surface, the ears were incubated in 70% ethanol for 5 min and then in 2.4% sodium hypochlorite supplemented with 0.1% Tween for 20 min, before being washed five times in sterile distilled water for 5 min each. Kernels of normal and healthy morphology were sampled for differentiation. Immature embryos were isolated with a lancet, and up to 30 were collected in a 2-mL tube containing 1 mL of sterile distilled water.

A type I callus, which are clumps of nonpolar cells with a smooth surface and a hard shell, were generated at the edge of the immature shield after 20–40 days on induction medium (Figure 4b). After 60–90 days, a type II callus with dense structure was induced. Further incubation led to the induction of an embryonic callus characterized by bright color, loose structure, granular shape, dry surface, and rapid growth. Callus can be classified into two groups, according to granulation and surface hardness, dense type II callus (Figure 4c), and loose type II callus (Figure 4d).

### 4.2. Media

Media preparations and related procedures were performed, as described previously [19], with the specific compositions being listed in Table 3. Established procedures were modified, as follows: (1) copper sulfate, acetosyringone, L-cysteine, and dithiothreitol were added to co-cultivation medium; (2) cefotaxime was replaced with carbenicillin in resting media; (3) co-cultivation, resting, and selection media were supplemented with silver nitrate; (4) basal salt and infection medium were autoclaved; and, (5) glucose, antioxidants, vitamins, and antibiotics were filter-sterilized.

### 4.3. Morphogenic Vector Design and Agrobacterium Strains

**Figure 5:**
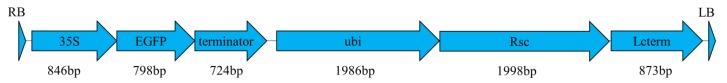
Organization of the binary plasmid used for *Agrobacterium*-mediated transformation. LB, left border; RB, right border. The *egfp* gene was used as a visual marker for selection and was driven by the CaMV35S (35S) promoter. The reporter regulator gene (*Rsc*) was controlled by the maize polyubiquitin gene (*ubi*) promoter.

Figure 5 shows a representation of the pHZM1-Rsc transformation construct; the plasmid was kindly provided by the National Key Laboratory of Crop Genetic Improvement, Huazhong Agricultural University. Two expression cassettes, CaMV35S:*egfp* and UBI:*Rsc*, were inserted into the *pEGAD* vector to generate the cloning vector.

The vector was compounded by Make Research Easy (Nanjing, China) and was transferred into *Agrobacterium tumefaciens* strain EHA105 by electroporation. The bacteria were precultivated for 2–3 days on LB solid medium with 100 mg/L kanamycin at 28 °C in the dark. On the day of transformation, *Agrobacterium* colonies were collected from the plate with a spatula, resuspended in N-I infection medium with 100 μM acetosyringone (Table 5), and incubated for 2–3 h at 28 °C and 200 rpm. Cell density, measured as OD_600_, was adjusted prior to embryo infection (see below).

### 4.4. Plant Transformation and Optimization of the Agrobacterium Transgenic System on Calli

Plant transformation followed an established standard protocol with some modifications [22]. Immature embryos and calli were collected in 10-mL microcentrifuge tubes containing N-I liquid medium with 100 μM acetosyringone and they were allowed to sit for 20 min. If using immature embryos as the receptor material, 30 immature embryos were placed into one 10-mL microcentrifuge tube, 30 tubes were infected at one time separately, and the experiment was repeated three times. The experiment was repeated three times for the setting of 3 g per tube of 30 tubes, when the callus was used as the recipient material for infection and transformation. The solution was drawn off, the calli were then infected with 5 mL of *Agrobacterium* suspension containing the pHZM1-Rsc binary vector, followed by brief vortexing for 5 min at 28 °C. The *Agrobacterium* liquid was drawn off, and the infected callus or immature embryos were transferred onto the solidified co-cultivation medium (Table 5). The embryos were placed with embryo axis side in contact with the co-cultivation medium and the calli were tiled on the medium. The plate was sealed with Parafilm and incubated in the dark at 19°C for three days. After co-cultivation, the callus and embryos were rinsed four times with sterilize distilled water, followed by two times (5 min) with sterile water containing 100 mg L^−1^ carbanicillin (ICN, Costa Mesa, CA, USA) and were blotted dry on a sterile filter paper (Whatman No. 1). They were then transferred onto the N-R resting medium (Table 5) containing 100 mg L^−1^ carbenicillin (ICN, Costa Mesa, CA, USA) and incubated at a temperature of 28 ± 2 °C for 7 days in the dark.

To optimize the *Agrobacterium* transgenic system on calli, the transformation conditions were analyzed by gradient method, including the concentration of bacteria solution, co-culture time, and the amount of acetyleugenone added [50]. The specific settings are as follows: (1) Bacterial concentration was set up at 0.2, 0.4, 0.6, 0.8, 1.0, and 1.2 at OD_600_ in the infection medium (N-I). The infection medium containing bacteria of different concentrations were selected and transfected according to the above transgenic process. (2) Bacterial concentration was set up at 1.0 (OD_600_) in the infection medium (N-I). The calli were transferred onto the solidified co-cultivation medium (Table 4) after the infection stage, and plates were sealed with Parafilm and incubated in the dark at 19 °C for one day to seven days, each additional day is treated as one condition. (3) Bacterial concentration was set up at 1.0 (OD_600_) in the infection medium (N-I), add 0 μM, 50 μM, 100 μM, 150 μM, and 200 μM acetyleugenone, respectively. The infection calli were transferred onto the solidified co-cultivation medium, incubated in the dark at 19 °C for five days.

After seven days restoration of culture, the instantaneous conversion efficiency was calculated by observing the green fluorescence performance on the DR-46B Dark Reader transilluminator (see below). Subsequently, the immature embryos and the calli were transferred to N-S medium (Table 5) to selection culture in the dark at 28 ± 2 °C. Two weeks after the first round of selection, tissues were transferred to fresh N-S medium and sub-cultured at 2–3 week intervals. After the third round of selection, the tissues were broken into small pieces and selected based on green fluorescence performance on the DR-46B Dark Reader transilluminator (see below).

After three round of selection, the fluorescence phenotypic in transformed maize calli were detected with a fluorescent protein macro detector set (Nikon, Tokyo, Japan), were transferred to a regeneration medium, and incubated at 25 °C under a 16/8 h (light/dark) photoperiod. Plantlets regenerated from the selected callus within 2–3 weeks were transferred to a tube containing rooting medium. Plantlets with fully-grown roots were transplanted into soil and grown under greenhouse conditions [52].

### 4.5. GFP Fluorescence Assay

The transformed maize calli were examined under a DR-46B Dark Reader transilluminator (Clare Chemical Research, Dolores, CO, USA) and a digital camera (a550; Sony, Tokyo, Japan) with a GFP filter. The instantaneous conversion efficiency was calculated by observing the green fluorescence performance on the DR-46B Dark Reader transilluminator after the resting culture. The statistical method was the proportion of immature embryos with green fluorescence in all immature embryos or the weight proportion of callus with green fluorescence callus. After three rounds of selection culture, the conversion efficiency was calculated observing the green fluorescence performance on the DR-46B Dark Reader transilluminator, the statistical method was same with the instantaneous conversion efficiency calculate.

The GFP fluorescence phenotypic in transformed maize calli was showed with a fluorescent protein macro detector set (Nikon, Tokyo, Japan) using an appropriate excitation source (460–495 nm) and filter (FHS/EF-2G2; 505–515 nm). Fluorescence images of calli in Petri dishes were obtained using a digital camera (a550; Sony, Tokyo, Japan) with a GFP filter.

### 4.6. Pretreatment with Lytic Enzyme Solution

Preparations of 1 g/mL of cellulase (Takara, Tokyo, Japan) and 1 g/mL pectinase (Takara, Japan), mixed in a 1:1 solution, and stored at 4 °C before use. The mixed enzyme solutions at different concentrations (0.000, 0.010, 0.020, 0.030, 0.040, and 0.050 g/mL) were diluted with infection medium at the time of treatment. After pretreatment for 5 min in infection medium containing different concentrations of enzyme solution, then rinsed five times with sterilize distilled water, and were blotted dry on a sterile filter paper (Sigma-Aldrich, Shanghai, China). The process of transformation and detection was in accordance with the optimized *Agrobacterium*-mediated transgenic system on callus.

At a designated concentrations point (0.030 g/mL), calli were pretreated by 0, 3, 6, 9, 12, and 15 min, separately. Rinsed five times with sterilize distilled water and were blotted dry on a sterile filter paper (Whatman No. 1) after the callus pretreatment. The process of transformation and detection was in accordance with the optimized *Agrobacterium*-mediated transgenic system on callus.

Two treatment conditions were used for two-column hybridization, and the experimental gradient was set, respectively, mixed enzyme solutions concentrations (0.000, 0.010, 0.020, 0.030, 0.040, and 0.050 g/mL) and pretreated time (0, 3, 6, 9, 12, and 15 min). Rinsed and transfered in accordance with the optimized *Agrobacterium*-mediated transgenic system on callus.

At least three microcentrifuge tubes were prepared per experiment, and every tube contained 3 g callus that had been washed twice with infection medium containing acetosyringone (100 μM) and 1.0 (OD_600_) bacterial concentration. The instantaneous conversion efficiency and the conversion efficiency were observed based on green fluorescence performance on the DR-46B Dark Reader transilluminator.

### 4.7. Transgene Analysis

Putative transgenic plants were subjected to molecular analysis to confirm the presence of the Rsc reporter. Genomic DNA was extracted from young leaves of transgenic and non-transgenic plant lines at the adult stage using DNA extraction buffer (100 mM Tris-HCl pH 8, 10 mM EDTA pH 8, and 1 M KCl). A pair of primers was designed for PCR analysis: RscF (5′-GGGTTTAGGGTTAATGGT-3′) and RscR (5′-CACTGGCAAGTTAGCAAT-3′). The expected *Rsc* PCR product contained part of the sequence of the *ubi* promoter and part of the *Rsc* gene. The cycling parameters for *Rsc*-PCR were as follows: 94 °C for 5 min; 33 cycles of 94 °C for 30 s, 58 °C for 30 s, and 72 °C for 2 min; and, 72 °C for 5 min. The amplification product (914 bp) was checked on 0.8% agarose gel and photographed [28].

## 5. Conclusions

The present study describes how we successfully improved *Agrobacterium*-mediated gene delivery in maize using an embryonic callus and a pretreatment protocol. This strategy has allowed us to dramatically shorten, from eight to three months, the time it takes to obtain maize transgenes. The resulting transformants should be considered representative, because the calli we used were from various growing seasons. Given that our improved protocol utilizes standard binary constructs and thus does not require a super-binary vector, this improved gene delivery system will be instrumental for public laboratories to study both basic and applied plant biology in transgenic maize. The maize transformants that were recovered in this study may represent a fraction of transgenic cells that survived lethal events. Conceivably, other important maize engineering efforts, such as functional genomic studies, will also benefit from this high-frequency transformation process.

## Figures and Tables

**Table 1:** The instantaneous conversion efficiency in different types of receptor materials in three repeats.

Material	Receptor Number	GFP Positives	GFP Positives	GFP Positives	Average	Standard Deviation
Immature Embryos	900	40	67	38	0.053	0.018
Type I Callus	90 g	6.3 g	3.51 g	2.88 g	0.047	0.021
Dense Type II Callus	90 g	5.94 g	5.67 g	4.32 g	0.059	0.010
Loose Type II Callus	90 g	4.23 g	5.22 g	5.49 g	0.055	0.007

**Table 2:** The conversion efficiency and loose causes in different types of receptor materials.

Material	Conversion Efficiency (g)	Callus Induction Loose (g)	*Agrobacterium* Infection Loose (g)
Immature Embryos	0.05 ± 0.04	5.24 ± 1.78	0.01 ± 0.01
Type I Callus	0.08 ± 0.02	1.15 ± 0.20	3.47 ± 2.07
Dense Type II Callus	0.69 ± 0.04	0.18 ± 0.03	5.03 ± 0.93
Loose Type II Callus	4.72 ± 0.50	0.19 ± 0.03	0.86 ± 0.64

**Table 3:** Effect of pretreatment conditions on *egfp* expression.

Mixed Enzyme Solution Concentration (g/mL)			Mixed Enzyme Treatment Time (min)		
Concentration	Frequency of Callus Rate	Frequency of Stable Transformation	Time	Frequency of Callus Rate	Frequency of Stable Transformation
0	73.19 ± 3.51	15.57 ± 1.89	0	76.46 ± 3.21	15.22 ± 0.60
0.01	64.50 ± 3.06	22.67 ± 1.42	3	71.00 ± 2.00	20.70 ± 0.31
0.02	53.56 ± 3.60	29.37 ± 1.96	6	63. 03 ± 8.66	25.02 ± 0.28
0.03	48.56 ± 3.60	37.48 ± 0.49	9	53.75 ± 1.52	38.33 ± 0.56
0.04	34.59 ± 3.78	21.28 ± 1.22	12	42.89 ± 11.71	12.39 ± 1.28
0.05	18.47 ± 1.15	10.54 ± 0.99	15	43.05 ± 3.60	10.15 ± 0.52

Each test was performed three times, and the mean ± SD was used to calculate the %.

**Table 4:** Effect of combined changes in pretreatment conditions on *egfp* expression.

	0 min ^2^	3 min	6 min	9 min	12 min	15 min
0 g/m ^1^	15.57 ± 1.89 ^3^	21.17 ± 2.57	25.53 ± 3.10	39.23 ± 4.77	12.61 ± 1.53	10.27 ± 1.25
0.01 g/mL	22.67 ± 1.42	30.83 ± 1.94	37.18 ± 2.33	57.13 ± 3.58	18.36 ± 1.15	14.96 ± 0.93
0.02 g/mL	29.37 ± 1.95	39.95 ± 2.66	48.17 ± 3.20	74.02 ± 4.92	23.79 ± 1.58	19.39 ± 1.29
0.03 g/mL	37.48 ± 0.49	50.98 ± 0.66	61.47 ± 0.80	94.46 ± 1.23	30.36 ± 0.39	24.74 ± 0.32
0.04 g/mL	21.28 ± 1.22	28.95 ± 1.66	34.90 ± 2.00	53.63 ± 3.08	17.24 ± 0.99	14.05 ± 0.80
0.05 g/mL	10.54 ± 0.99	14.34 ± 1.35	17.29 ± 1.63	26.57 ± 2.51	8.54 ± 0.80	6.96 ± 0.65

^1^ Pretreatment concentration in g/mL; ^2^ pretreatment times in min; ^3^ frequency of interaction. Each test was performed three times, and the mean ± SD was used to calculate the %.

**Table 5:** Composition of media used in *Agrobacterium*-mediated transformations.

Medium	Composition
LB (solid)	Yeast extract 5 g/L, NaCl 10 g/L, peptone 10 g/L, agar 15 g/L, pH 6.8
LB (liquid)	Yeast extract 5 g/L, NaCl 10 g/L, peptone 10 g/L, pH 6.8
Infection (N-I)	N6^1^ 2 g/L, 2,4-D^1^ 2.0 mg/L, L-proline 0.7 g/L, sucrose 68.4 g/L, D-glucose^2^ 36 g/L, MES^1^ 0.5 g/L, myo-inositol 0.1 g/L, As^1,2^ 200 μM, pH 5.2
Co-cultivation (N-C)	N6 4 g/L, 2,4-D 2.0 mg/L, L-proline 0.7 g/L, sucrose 30 g/L, MES 0.5 g/L, myo-inositol 0.1 g/L, CuSO_4_^1,2^ 0.05 µM, DTT^1,2^ 1 M, L-cysteine 0.4 g/L, As 100 μM, agar 8 g/L, pH 5.8
Resting (N-R) Selection (N-S) Regeneration (MS-D) Rooting (MS-R)	N6 4 g/L, 2,4-D 2.0 mg/L, L-proline 0.7 g/L, sucrose 30 g/L, MES 0.5 g/L, myo-inositol 0.1 g/L, AgNO_3_^1,2^ 0.85 mg/L, carbenicillin^1,2^ 0.1 g/L, gelrite 2.5 g/L, pH 5.8 Resting medium without carbenicillin, pH 5.8 MS^1^ 4.3 g/L, sucrose 30 g/L, myo-inositol 0.1 g/L, 6-BA 3.5 mg/L, gelrite 3.0 g/L, pH 5.8 MS 4.3 g/L, sucrose 25 g/L, NAA 0.5 mg/L, gelrite 2 g/L, pH 5.8

^1^ 6-BA, 6-Benzylaminopurine; N6, Chu medium salt with N6 vitamins; 2,4-D, 2,4-dichlorophenoxyacetic acid; MES, 2-(N-morpholino) ethanesulfonic acid; As, acetosyringone; CuSO_4_, copper sulfate; DTT, dithiothreitol; AgNO_3_, silver nitrate; MS, MS basal salt and vitamins; NAA,. ^2^ Components were filter sterilized.
